# The Effects of Two Intervention Strategies to Reduce the Intake of Salt and the Sodium-To-Potassium Ratio on Cardiovascular Risk Factors. A 4-Month Randomised Controlled Study among Healthy Families

**DOI:** 10.3390/nu12051467

**Published:** 2020-05-19

**Authors:** Ulla Toft, Nanna Louise Riis, Anne Dahl Lassen, Ellen Trolle, Anne Helms Andreasen, Amalie Kruse Sigersted Frederiksen, Niklas Rye Joergensen, Jens Kristian Munk, Kirsten Schroll Bjoernsbo

**Affiliations:** 1 Center for Clinical Research and Prevention, Bispebjerg and Frederiksberg Hospital, 2000 Frederiksberg, Denmark; nanna.louise.riis@regionh.dk (N.L.R.); anne.helms.andreasen@regionh.dk (A.H.A.); amalieksf@gmail.com (A.K.S.F.); kirsten.bjoernsbo@regionh.dk (K.S.B.); 2 Department of Public Health, Faculty of Health and Medical Sciences, University of Copenhagen, 2100 Copenhagen, Denmark; 3 National Food Institute, Technical University of Denmark, 2800 Kgs. Lyngby, Denmark; adla@food.dtu.dk (A.D.L.); eltr@food.dtu.dk (E.T.); 4 Department of Clinical Biochemistry, Rigshospitalet, 2100 Copenhagen, Denmark; niklas.rye.joergensen@regionh.dk; 5 Department of Clinical Medicine, Faculty of Health and Medical Sciences, University of Copenhagen, 2100 Copenhagen, Denmark; 6 Department of Clinical Biochemistry, Amager and Hvidovre Hospital, 2650 Hvidovre, Denmark; jens.kristian.munk@regionh.dk

**Keywords:** salt reduction, potassium, families, real life clinical trial, bread reformulation, cardiovascular risk factors

## Abstract

The aim was to examine the effects of two different salt reduction strategies on selected cardiovascular risk factors. The study was a four-month cluster randomised controlled study. Eighty-nine healthy Danish families (309 individuals) were randomly assigned to either (A) gradually salt-reduced bread, (B) gradually salt-reduced bread and dietary counselling to further reduce salt intake and increase potassium intake or (C) standard bread (control). The effect was assessed using linear mixed models. Intention to treat analyses comparing changes in the three groups showed a significant reduction in body fat percent (−1.31% (−2.40; −0.23)) and a borderline significant reduction in total plasma cholesterol (−0.25 mmol/L (−0.51; 0.01) and plasma renin (−0.19 pmol/L (−0.39; 0.00) in group A compared to the control group. Adjusted complete case analyses showed a significant reduction in total plasma cholesterol (−0.29 mmol/L (−0.50; −0.08), plasma LDL cholesterol (−0.08 mmol/L (−0.15; −0.00)), plasma renin (−0.23 pmol/L (−0.41; −0.05)), plasma adrenaline (−0.03 nmol/L (−0.06; −0.01)) and body fat percent (−1.53% (−2.51; −0.54)) in group A compared to the control group. No significant changes were found in group B compared to the control group. In conclusion, receiving sodium reduce bread was associated with beneficial changes in cardiovascular risk factors. No adverse effects were observed.

## 1. Introduction

Excessive dietary salt intake has repeatedly been found to have a major role in the pathogenesis of hypertension, the leading risk for premature death in the developed and developing world [1,2,3]. Increasing evidence furthermore indicates that high salt intake directly increases the risk of cardiovascular disease (CVD) [4,5,6,7] and possible other non-communicable diseases such as gastric cancer, kidney stones, osteoporosis and obesity [3]. Based on the current evidence, excessive intake of salt has been estimated to be one of the leading risk factors for disease and mortality worldwide [6].

The dietary salt intake in most countries, is far beyond the recommended level (<5–6 g/day) [8,9] and population-based reduction in salt consumption has been rated to be one of the most cost-effective strategies to prevent cardiovascular disease (CVD) [10,11]. Therefore, several countries have initiated national programs to decrease salt intake in the population [12].

When introducing public health strategies like population-based salt reduction, it is crucial to ensure that these are supported by sound scientific evidence. In addition, it is important to investigate potential adverse effects of the strategies. Hence, some randomised studies have shown adverse effects of lowering the daily salt intake on levels of blood lipids, renin, aldosterone and catecholamines and thereby indicated a potential adverse effect of low salt intake on CVD [13]. However, these findings were mainly based on very short-term studies (a few weeks) with a large acute salt reduction. Thus, there is a need for studies that explore these mechanisms during a modest reduction in salt intake for a longer period analogously to the current public health recommendation of gradual salt reduction [2].

Most national programs in high-income countries with the aim to reduce salt intake on a population-based level rely mainly on food product reformulation as up to 80% of the salt intake in western countries comes from salt added to processed foods [14]. The biggest contributor of salt intake in most of these countries is bread [15]. Therefore, reducing the salt content in bread could be a relevant part of an effective strategy to reduce the salt intake of the population. As a sudden, large reduction of salt could/would/might make foods unacceptable to consumers [16], a gradual reduction of the salt content is generally recommended [9].

Besides reformulation of foods, changes in dietary habits and practices among consumers might be necessary in order to reach the recommended level of salt intake. Furthermore, potassium has been found to potentially attenuate some of the negative effects of a high salt intake [17,18,19,20] and some earlier studies have found that a high sodium/potassium ratio is a stronger risk factor for elevated blood pressure and cardiovascular disease than levels of either salt or potassium alone [18]. Hence, a strategy combining food reformulation and dietary counselling is potentially the most efficient strategy to reach the recommended daily level of salt intake and simultaneously increase the intake of potassium and thereby further improve the health impact. The role of potassium is however not yet well understood [20], and the evidence regarding the effect of different salt reduction strategies on cardiovascular risk factors in real life settings and among healthy families is very sparse.

The aim of this paper was to examine the effects of the two different salt reduction strategies on selected cardiovascular risk factors. Our hypothesis was to see a greater effect on cardiovascular risk factors when combining reformulation with dietary counselling compared with reformulation alone. Furthermore, we expected to see positive effects on blood pressure whereas it was less clear from the literature if there would be adverse effect of salt reduction on, e.g., plasma lipid and plasma hormones.

## 2. Materials and Methods

The SalT Reduction InterVEntion (STRIVE) study was registered as a clinical trial at Clinical Trials.gov (https://clinicaltrials.gov/, trial number NCT03810885). The study has been conducted in accordance with the Helsinki Declaration guidelines and has been approved by the Danish National Ethics Committee (approval number: H-17030995) and the Danish Data Protection Agency. The STRIVE study is described in brief below, including relevant details on participants, materials, methods and design which have been described previously [21,22]; however, this information is necessary in order to understand the results from this study.

### 2.1. Participants

Participants were recruited as families from five municipalities (Albertslund, Ballerup, Egedal, Glostrup and Rødovre) in the Southwestern part of the Capital Region of Denmark. These municipalities were all located close to the research centre or the bakery producing the intervention bread. Families were recruited through social media at schools, kindergartens and large companies, word of mouth and posters in the local area during the period January–February 2018. Inclusion criteria were family with at least one child (3–17 year) and one parent (18–65 year) and a daily bread consumption among adults. Children of divorced parents had to stay more than half the time with the participating parent, if both parents were not participating. Exclusion criteria were antihypertensive and lipid-lowering treatment, pregnancy, diabetes, coronary heart disease and urine albumin > 300 mg/day.

Written informed content was obtained from all participants ≥ 18 years and primary caregivers for participants < 18 years before participation.

### 2.2. Study Design and Intervention

The STRIVE study was a four-month, single-blinded, cluster randomised controlled trial with a parallel design. The families included were randomly allocated, using a computer-generated sequence of random group assignment, into one of three groups receiving either (1) salt reduced bread (intervention A), (2) salt reduced bread and dietary counselling (Intervention B) or (3) bread with regular salt content (control group). Allocation was performed after the clinical measurements at baseline. Two families included three parents, as divorced parents’ new partners were included. Blinding of participants was achieved through color-coding of bread packaging.

Baseline measurements were conducted from 1 February to 6 March 2018, after which families began to receive the bread from the study. The families collected the bread free of charge twice a week at the bakery (Mondays and Thursdays) or at the research centre, depending on preference. Participants were instructed to replace their usual consumption of bread with the bread products provided in the study, and the amount was adjusted to fit the usual bread habits within the family. The bread products constituted of a mixture of rye bread and wheat bread formed as a loaf or as buns. At each bread hand-out the families were provided with rye bread, loaf and buns, with different recipes used for the loaf and the buns. The rye bread and either the loaf or the buns fulfilled the keyhole label criteria for wholegrain, which is at least 35% wholegrain for rye bread and 30% wholegrain for wheat bread [23]. During the first two weeks of intervention, the sodium content in the bread was 0.48 g/100 g (1.2 g salt/100 g), which was maintained in the control group during the entire intervention. Within the two intervention groups, this was gradually reduced by 0.08 g/100 g (0.2 g salt/100 g) each week until a sodium content of 0.24 g/100 g (0.6 g salt/100 g) in rye bread and 0.16 g/100 g (0.4 g salt/100 g) in wheat bread was reached, which was maintained during the rest of the intervention period. During the gradual sodium reduction, some of the regular sodium chloride (salt) was replaced with viva salt. Viva salt is naturally low in sodium and was added to enhance the salt taste slightly. The sodium content in bread was tested every week by the Danish Veterinary and Food Administrations accredited laboratory using the ICP-OES method, which is accredited by the Danish Accreditation Fund (DANAK) according to ISO 17025.

In addition to the salt reduced bread, a dietary advice program was given to intervention group B. The program was developed to reduce dietary salt and increase dietary potassium. A thorough description has been provided elsewhere [21,22]. In brief, the dietary counselling included five main messages: (1) buy less foods rich in salt within various food categories, (2) eat less foods with a high salt content, (3) reduce the use of salt during cooking and at the table, (4) flavour food without using salt using other herbs and spices and (5) follow the plate model to increase the intake of fruit and vegetables.

The dietary counselling consisted of a 2-h group introduction, a 1-h family counselling session, followed by two telephone counselling sessions with one of the parents and weekly e-mails during the entire intervention period. In order to measure compliance, missed bread hand-outs were recorded, and all family members were asked to register their daily intake of intervention bread in compliance sheets given to the families on a weekly basis. Participants were regarded as compliant, if they collected 80% of the intervention bread and reported consumption of the intervention bread in the returned sheets at least 80% of the days. Additionally, participants in intervention B had to participate in the individual family counselling and at least one follow-up telephone counselling to be regarded as compliant.

### 2.3. Outcome Measurements

Families visited the research centre (Centre for Clinical Research and Prevention (CCRP)) at baseline and at four-month follow-up for a health examination.

Height was measured without shoes to the nearest cm, weight without shoes and coat to the nearest kg, and body mass index (BMI) was calculated (kg/m^2^). Body fat percentage was determined from impedance.

Blood pressure (BP) was measured thrice with an electronic blood pressure monitor (Medidyne) after 5 min of rest in sitting position. Systolic and Diastolic BP was calculated as the average of the last two measurements.

All adults and children from the age of 10 were asked to be fasting a minimum of two hours before arrival at CCRP. Blood samples were collected after 30 min at complete rest in lying position and were optional in children 10–17 years old and mandatory in adults, whereas blood samples were not collected for children below the age of ten.

Plasma total and HDL-cholesterol, triglyceride and glucose were measured using colorimetric slide test (Vitros 5.1, Ortho Clinical Diagnostics, Raritan, NJ, USA). VLDL- and LDL-cholesterol were calculated by Friedewald’s equation. HbA_1c_ in plasma were determined using a chemiluminescence assay on the automated analyser iSYS (Immuno Diagnostic Systems, Boldon, Tyne and Wear, UK), and adrenalin and noradrenalin were measured by analyses of methoxycatecholamines by liquid chromatography-mass spectrometry. Aldosterone and renin were sampled in EDTA glasses, centrifuged and plasma stored for 4–8 months at −80 °C before analysis by chemiluminescent immunoassay.

To estimate daily salt and potassium intake participants were asked to collect 24-h urine samples. Parents and children >18y were instructed to collect three consecutive 24-h urine samples, preferably one weekend day and two weekdays, whereas children <18 year were instructed to collect one 24-h urine sample on a weekend day. Completeness was validated using the PABA method for adults (for more details see [22]).

### 2.4. Covariates

At baseline participants answered a questionnaire on gender, age, marital status, occupation, education, health (diagnosed with cancer, diabetes, hypertension, high cholesterol, myocardial infarction, stroke and or coeliac disease) and lifestyle (physical activity, smoking, alcohol and dietary habits) (described in detail elsewhere [22]).

### 2.5. Statistical Analyses

Power calculation was based on the primary outcome, salt intake measured by repeated 24-h urine sodium content, taking the cluster-randomised design into account. The expected reduction in the estimated salt intake when consuming the salt reduced bread was 1.25 g/day (0.5 g sodium/day), and in combination with dietary counselling, the average reduction was expected to be 3 g/day (1.2 g sodium/day) (for details see Reference [19]). Population-based studies conducted at Centre for Clinical Research and Prevention identified an average salt intake of 8.3 g, Std 2.2 (3.3 g sodium/day) among adults. A family was assumed to consist of four members, and the intra-class correlation within families was assumed to be 0.33. A total of 25 families (100 participants) in each of the three groups was needed to identify differences in salt intake of 1.2 g/day (0.5 g sodium/day), at a 5% level and with 80% power.

Categorical data are presented as frequencies and proportions and continuous variables are presented as mean ± std or median and interquartile range (IQR) when appropriate according to distribution. All baseline values are presented based on the observed values. BMI, triglycerides, HDL-, LDL- and VLDL cholesterol, aldosterone, renin and plasma-glucose was log-transformed before analyses to meet assumptions of normal distribution.

The effect of the intervention on cardiovascular risk factors was evaluated using “intention to treat” analyses, including all participants regardless of the adherence to the intervention and dropouts. To create a full analysis data set, missing data at baseline and follow-up were imputed using multiple imputation with 100 samples and taking into account cluster effects. To test the effect of the intervention, linear mixed model with the cardiovascular risk factors at follow up as outcome variable, treatment group and baseline measure of the risk factor as fixed effects and family ID as random effect were used. The variance structure was chosen as compound symmetry unless a structure with different covariance for adults and children had a better fit (based on AIC) on a dataset consisting of complete cases (before multiple imputation). Adjusted analyses were furthermore done adjusting for sex, age, BMI, education and physical activity. Analyses for all participants for blood pressure were furthermore adjusted for alcohol intake. Development in the three groups were compared pairwise within the overall analyses.

Furthermore, complete case analyses were conducted including only participants with at least one measurement at both baseline and follow-up. Complete case analyses were adjusted for sex, age, BMI, education and physical activity. Analyses for all participants for BMI and blood pressure were furthermore adjusted for alcohol intake.

Subgroup analyses were performed investigating the effect of reducing estimated salt intake in the intervention groups with at least 20% and the sodium to potassium ratio with at least 20%, respectively, on changes in the selected cardiovascular risk factors.

The imputations were carried out using R version 3.4.1 (Jomo package). All other analyses were carried out using SAS 9.4 TSM5 (SAS Institute Inc). Levels of significance were set at 0.05 for all analyses.

## 3. Results

**Figure 1:**
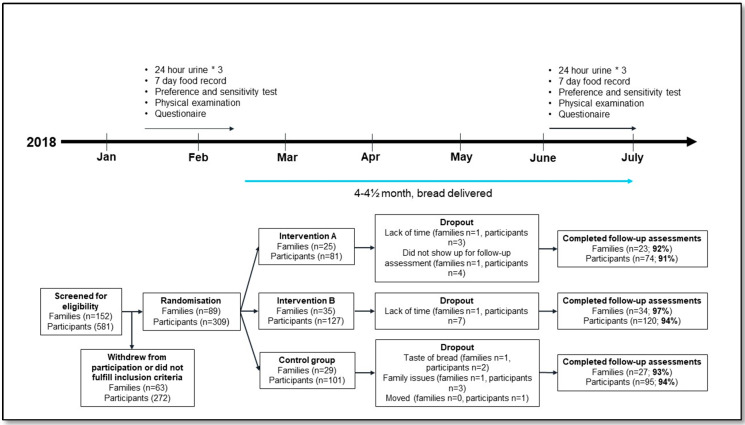
Design and flowchart.

A total of 152 families were screened for eligibility. Out of these, 91 met the inclusion criteria and agreed to participate in the study. During the baseline health examination, five participants were excluded for not meeting the inclusion/exclusion criteria. Two of the excluded participants were the only children between 3–17 years in their families, which is why the rest of the family was also excluded. A total of 89 families (309 persons) were included in the study. During the intervention, five families and two persons from other families (n = 20) dropped out. A flowchart of the study is shown in Figure 1. A total of 84 families (289 persons) completed the study.

Baseline characteristics of included families are shown in Table 1. The computer-generated sequence of random group assignment by chance included more families to intervention B, which is why the number of participants in this group is also higher than in the two other groups. The distribution of adults and children, family size, sex and age seemed balanced between the three groups, while weight, height and BMI seemed slightly lower in the control group compared to the two intervention groups. More participants in intervention B were vigorously active while fewer were moderately active, and in intervention A, more participants occasionally consumed alcohol.

Table 2 shows the mean (std)/median (IQR) and the unadjusted development in cardiovascular risk factors from baseline to follow-up in each group. Significant reduction on both diastolic blood pressure (DBP) and systolic blood pressure (SBP) were found in all three groups. Plasma Noradrenaline and body fat percent decreased significantly in both intervention groups but not in the control group. Plasma total cholesterol decreased significantly in group A whereas the plasma renin level and HbA1c increased significantly in group A and the control group. No significant changes were found for BMI, adrenalin, plasma triglycerides, HDL cholesterol, LDL cholesterol, VLDL cholesterol, aldosterone and glucose.

The primary outcome measure in this study was changes in salt intake, measured by sodium content in repeated 24-h urine samples, which is reported elsewhere [21]. The results showed a significant decrease in salt intake (g/day) in intervention group B and a non-significant decrease in intervention group A compared to the control group [21]. Furthermore, a significant decrease in the sodium to potassium ratio in intervention group B and a non-significant decrease in group A compared to the control was found [21].

Results from the intention to treat analyses comparing the three groups and using multiple imputation are shown in Table 3. No significant changes were found in group B compared to the control group. A significant reduction in body fat percent (−1.3% (−2.40; −0.23)) and a borderline significant reduction in total plasma cholesterol (−0.25 mmol/L (−0.51; 0.01)) and plasma renin (−0.19 pmol/L (−0.39; 0.00)) were found in group A compared to the control group. Comparing the two intervention groups, renin significantly increased and HbA1c decreased in group B compared to group A. The analyses were furthermore adjusted for sex, age, BMI, physical activity, alcohol (only for blood pressure) and education (data not shown). Similar results were found, hower adjusted analyses showed a significant reduction in total plasma cholesterol in group A compared to the control group (−0.28 mmol/L (−0.54; −0.02)).

Adjusted complete case analyses (Table 4) showed a significant reduction in total plasma cholesterol (−0.29 mmol/L (−0.50; −0.08), plasma LDL cholesterol (−0.08 mmol/L (−0,15;−0.03)), plasma renin (−0.23 pmol/L (−0.41; −0.05)), plasma metanephrine (adrenaline) (−0.03 nmol/L ((−0.06; −0.01)) and body fat percent (−1.5% (−2.51; −0.54)) in group A compared to the control group. No significant changes were found in group B compared to the control group. Compared to group A, the plasma renin level increased significantly (0.24 [0.06; 0.41]) and HbA1c decreased significantly in group B. No significant effects were found for DBP and SBP, plasma triglyceride, HDL cholesterol, aldosterone, noradrenaline and BMI.

Table 5 shows results from subgroup analyses among participants in the intervention groups that decreased their estimated daily salt intake by at least 3 g or the sodium to potassium ratio by at least 20% from baseline to 4-month follow-up. For those that reduced their salt intake by at least 3 g per day from baseline to follow-up, a significant decrease in DBP and SBP, plasma total cholesterol, adrenalin and noradrenalin was found. Among those that reduced the sodium to potassium ratio by at least 20% a significant decrease was found for DBP and SBP, plasma total cholesterol, LDL cholesterol, VLDL cholesterol, noradrenalin and body fat percent. No significant changes were found for BMI, plasma triglycerides, HDL cholesterol, aldosterone, renin, glucose and HbA1c.

## 4. Discussion

The present study showed significant beneficial effects of providing gradual salt-reduced bread to healthy families during a four-month period. Hence, a significant reduction in total plasma cholesterol, plasma LDL cholesterol, plasma renin, plasma adrenaline and body fat percent were found compared to the control group (complete case analyses). Additional dietary counselling to decrease salt intake and increase the intake of potassium did not show any effect on cardiovascular risk factors. However according to subgroup analyses, a reduction in the sodium to potassium ratio of at least 20% was associated with beneficial changes in SBP and DBP, plasma cholesterol, noradrenaline and body fat percent. Similar results were found in the subgroup that reduced their estimated salt intake by at least 3 g/day. No adverse effects of the intervention were found.

Several previous studies have investigated the effect of salt reduction on SBP and DBP. A recent systematic review of randomised trials showed a dose response-relation between salt intake and blood pressure [24]. Each 50 mmol reduction in 24-h urinary sodium excretion was associated with a 1.10 mmHg reduction in systolic blood pressure and 0.33 mmHg reduction in diastolic blood pressure. In general, a smaller effect of salt reduction was found in normotensive and young people. The present study was conducted among healthy families with children below 18 years. Hence the expected effect on blood pressure is lower than average. A significant reduction in estimated salt intake of 1.44 g salt was found among adults in the intervention group B (combined salt reduced bread and dietary counselling) [21]. Due to low statistical power and a rather small reduction in measured salt intake, no significant effect was found on blood pressure, although a non-significant tendency of a reduced SBP (−1.50 mmHg) and DBP (−0.81 mmHg) was shown. These findings are in accordance with a comparable family-based cluster randomised controlled trial in Japan involving cooking classes given to housewives [25]. The study included 35 healthy housewives and 33 family members. Salt intake was reduced by 1.2 g per day on average. No significant effect was found on blood pressure.

Another recent comparable intervention study was conducted by Cashman et al. [26]. The intervention included 97 Irish adults with mildly to moderately elevated blood pressure, in a cross-over intervention design. The intervention consisted of the provision of salt-reduced bread, luncheon meats with no added salt, no-salt margarine and high-potassium fruit and vegetables drinks in addition to dietary counselling to reduce salt intake. Salt intake was reduced by 1.7 g on average, and SBP was significantly reduced by 3.3 mmHg whereas no significant effect was found for DBP. The greater effect on SBP in the study by Cashman et al. may partly be explained by the difference in blood pressure level at baseline. Hence Cashman et al. included only participants with elevated blood pressure [26].

He et al. did a large school-based education program to reduce salt intake among children and their families in China [27]. The children were educated how to reduce salt intake and delivered the messages to their families. Compared to the control group, the mean reduction in salt intake among children was 1.9 g/day and among adults −2.9 g/day. A significant effect on SBP was found among adults (−2.3 mmHg) but not among children. Hence, like the present study, there was a lower effect among children although the tendency was the same as among adults. However, the effect on salt intake among children was higher than the present study. This might be due to the fact that most of the salt in the Chinese diet is added by consumers in contrast to Denmark, where most of the salt is consumed through processed food whereby dietary counselling/teaching might be more effective in China than in Denmark. In the present study, we only provided salt reduced bread. A larger effect on salt intake and blood pressure would have been expected if more salt-reduced food groups had been provided to the participants.

Several previous intervention studies have investigated the relation between potassium intake and blood-pressure. A systematic review by Filippin et al. investigated the effect of potassium supplementation among the hypertensive [28]. Overall, potassium supplementation was found to decrease systolic blood pressure by 4.48 mmHg (95% CI 3.07–5.90) and diastolic blood pressure by 2.96 mmHg (1.10–4.82). The effect of increasing dietary potassium and decreasing dietary sodium to potassium ratio is much less investigated. In the present study, there was a significant effect on the sodium to potassium ratio [21] measured by 24-h urine in the combined intervention group (B). However, no effects on cardiovascular risk factors including blood pressure were found compared to the control group. In subgroup analyses including only participants that decreased the sodium to potassium ratio by at least 20%, significant beneficial associations were found with several risk factors including SBP and DBP, total plasma cholesterol, plasma LDL cholesterol, plasma VLDL cholesterol, plasma noradrenaline and body fat percent. These findings are, however, likely to be partly explained by other simultaneous dietary changes, e.g., an increase in the intake of vegetables, and might not exclusively be due to the reduction in sodium to potassium ratio per se.

While there is little disagreement that salt reduction is effective in reducing blood pressure and increasing evidence of the beneficial effects of increasing intake of potassium, especially among hypertensive, concerns have been raised by some researchers that salt reduction might lead to possible adverse effects in health. Especially the effect of salt reduction on plasma lipids, catecholamines, renin and aldosterone have been heavily debated. A systematic review by Graudal et al. [29] estimated the effects of low sodium diet versus high sodium diet on blood pressure, renin, aldosterone, catecholamines, cholesterol and triglyceride. Sodium reduction was found to result in a significant decrease in SBP and DBP but also a significant increase in renin, aldosterone, adrenaline, noradrenaline, cholesterol and triglyceride. However, Graudal et al. included a large number of very short-term trials with a large acute change in salt intake. A systematic review by He et al. [30] included only randomised trials with a modest reduction in salt intake and duration of at least 4 weeks to ensure a public health relevance. The results showed a significant increase in plasma renin, aldosterone and catecholamines but no effect on plasma lipids [30]. Aburto et al. [3] conducted a similar systematic review and meta-analysis including only studies among non-acutely ill adults, with a duration of at least four weeks and measure of 24-h urinary sodium excretion. Aburto et al. found no adverse effect on blood lipids and catecholamines.

To our knowledge, the present study is the first randomised controlled study that investigated the effect of a modest long-term salt reduction intervention among healthy families in a real life setting on levels of plasma renin, aldosterone, catecholamines and lipids. At 4-month follow-up no adverse effects on these cardiovascular risk factors were found. In contrast, subgroup analyses investigating the effect of salt reduction of at least 3 g per day showed significant beneficial effects on total plasma cholesterol, plasma adrenalin and noradrenaline. This could be explained by the body’s ability to adapt to a new level of salt intake when modest gradual changes are introduced during a longer time period, similar to what would be expected from the public health salt reduction initiatives. The increases in the above-mentioned risk factors are typically seen in studies of two or less weeks’ duration, including very large changes in salt intake [3]. When salt intake is reduced, blood volume reduces which activates the renin-angiotensin-aldosterone and sympathetic nervous systems [31]. However, long-term studies, including the present study, suggest that these changes are not lasting or might not occur with gradual and modest changes in salt intake.

Observational studies have indicated that excess salt intake may be an independent risk factor for obesity [32]. It has also been hypothesized that excess salt intake, especially among children and adolescents, is a determinant of sugar-sweetened soft drink consumption which increases the risk of obesity [32]. Evidence from randomised controlled trial (RCT) studies is however needed to confirm these findings. In the present study we found no effect of salt reduction on BMI, which was expected due to the modest reduction in the estimated salt intake. However, unexpectedly, a significant effect on body fat percent was found in group A. The reason for this will be explored in future analyses investigating possible dietary changes during the salt reduction intervention.

Systolic and diastolic blood pressure was reduced from baseline to follow-up both in the intervention groups and the control group. The reduced blood pressure in the control group can probably be explained both by the natural seasonal variation from winter to summer [33] but also by the fact that the salt content in the control bread was lower than the salt content in the usual bread sold by the baker [22]. Due to ethical considerations, we chose that the control bread should meet the criteria for healthy products (the keyhole label [22]) set by the health authorities. Hence, this is likely to have made the difference between the control and intervention groups smaller than if we had chosen to provide the usual high-salt bread from the bakery.

The effect on cardiovascular risk factors in this study was surprisingly higher in group A, who were provided only with salt reduced bread in contrast to intervention group B, who also received thorough dietary counselling on how to reduce salt intake and increase the intake of potassium. We have previously shown that the salt reduction in group B was higher than in group A. The reformulation intervention strategy in group A makes it possible to investigate the exclusive effect of salt reduction in a randomised single-blinded design, without changing other dietary factors. This is often not possible in dietary studies. Hence, the conclusions on the specific effect of salt reduction on cardiovascular risk factors are very strong in this design, although the salt reduction is lower than in the combined intervention. It might be speculated that, although group B reduced their salt intake more than group A, group B might have made some dietary changes that have had some adverse effects. This will be further explored in a future paper investigating dietary changes measured by 7-day food records in the STRIVE Study.

Missing data are unavoidable and can potentially lead to bias and incorrect conclusions. As recommended in the current scientific literature, we reported results of both complete-case (CC) and multiple imputation (MI) analyses [34,35]. MI are increasingly considered to be superior to CC methods in dealing with missing data. Hence, especially if missing data levels are high, it is well-documented that CC analyses may yield biased estimates and lead to reduced statistical efficiency. However, results from some recent simulation studies have found that if outcomes can be assumed as missing at random (MAR) or missing completely at random (MCAR), CC analyses were found to provide unbiased results and achieved precision similar or better than MI [34,35]. In this study MI analyses were used as the primary method for the intention-to-treat analyses whereas CC analyses were used for per protocol analyses. Results showed similar estimates; however, the statistical efficiency seemed to be lower in the MI analyses compared to CC. This contrasts with what might be expected because the sample size is lower in the CC analyses. This finding is however in accordance with the findings of earlier studies [34,35]. Is has been speculated that this may be due to an increase in the variability of the outcome, which again will affect the standard errors of the estimates [35]. The validity of the conclusions based on these analyses is however dependent on the degree and reasons for missing data. Of special interest is data missing at follow-up. In the present study, the participant rate at follow-up was high (91–97%). For the five families that dropped out during the intervention period, reasons were “lack of time” (n = 2), “the taste of the bread” (n = 1), “family issues” (n = 1), and one family just did not show up for the examination. Drop out was equally distributed between groups. Data on some of the outcome variables were furthermore missing due to difficulties in analysing these in the laboratory (especially Normetanephrine and Metanephrine). The later data are assumed to fulfil the criteria for MCAR whereas the dropout at follow-up is assumed to be missing at random (MAR), and hence, drop-out might be dependent on the included covariates (age, sex, BMI, education, physical activity) but independent of the unobserved outcome value. Based on these considerations, the conclusions based on both the MI and CC analyses in this study are assumed to be valid.

Some strengths and limitations of the present study need to be addressed. Important strengths of the study are the randomised controlled design, the thorough measurement of cardiovascular risk factors and that dietary salt was estimated based on repeated 24-h urine collections. Furthermore, an important strength of the study is the real-life setting in which the intervention took place, as the results can be translated into dietary behaviours in the general population.

Important limitations are the low statistical power due to a lower number of participants than planned, which were needed according to the power calculations, and the low level of salt reduction in the intervention groups. Therefore, it was not possible to investigate the potential adverse effect of larger salt reduction in a randomised controlled design. However, we investigated the effect of a salt reduction of at least 3 g/day on the intervention groups and did not find any adverse effect. Rather, we found several beneficial effects on the measured risk factors. A confounding factor in these analyses could be changes in other dietary factors. This paper did not include dietary data, but this will be further explored in a future paper including data from the 7-day food record.

In conclusion, providing gradual salt-reduced bread was associated with significant beneficial effects on changes in cardiovascular risk factors, whereas no significant effects were seen for the combined intervention (salt-reduced bread + counselling). A reduced salt intake (≥3 g per day) and a reduced sodium to potassium ratio were also found to be associated with beneficial changes in cardiovascular risk factors. No adverse effects were observed.

## Figures and Tables

**Table 1:** Baseline characteristics at cluster- and individual level ^a^.

	Intervention A	Intervention B	Control
***Cluster level***			
Families (n)	25 (28.1)	35 (39.3)	29 (32.6)
Participants (n)	81 (26.2)	127 (41.1)	101 (32.7)
Family size	3.2 ± 0.8	3.6 ± 1.1	3.5 ± 1.1
Parental education			
Short higher education < 3y or shorter ^b^ (n)	9 (37.5)	8 (24.2)	8 (28.6)
Medium higher education 3–4y (n)	6 (25.0)	12 (36.4)	7 (25.0)
Long higher education > 4y (n)	9 (37.5)	13 (39.4)	13 (46.4)
***Individual level***			
Children < 18y			
N	40	64	52
Sex (boys, %)	21 (52.5)	33 (51.6)	27 (51.9)
Age (y)	9.5 ± 4.2	9.1 ± 4.2	8.4 ± 3.5
Weight (kg)	40.3 ± 18.3	37.3 ± 19.6	32.0 ± 16.1
Height (cm)	144.1 ± 25.9	140.4 ± 27.4	133.1 ± 23.2
BMI (kg/m^2^)	18.0 ± 2.9	17.4 ± 2.8	16.9 ± 2.8
Physical activity			
Sedentary (n)	4 (10.0)	6 (10.0)	6 (12.0)
Moderately active (n)	7 (17.5)	10 (16.7)	6 (12.0)
Vigorously active (n)	29 (72.5)	44 (73.3)	38 (76.0)
Alcohol drinkers (n)	6 (15.0)	3 (5.0)	1 (2.0)
Smokers (n)	2 (5.0)	1 (1.7)	0 (0.0)
Salt intake (g/day) ^c^	6.2 ± 2.9	6.0 ± 3.4	5.5 ± 2.2
Sodium/potassium excretion ratio ^c,d^	2.2 [1.7,2.7]	2.2 [1.6,2.9]	2.4 [1.9,2.9]
Adults ≥ 18y			
N	41 (50.6)	63 (49.6)	49 (48.5)
Sex (men, %)	18 (43.9)	29 (46.0)	23 (46.9)
Age (y)	41.5 ± 9.5	40.5 ± 9.0	40.9 ± 8.0
Weight (kg)	78.6 ± 14.3	77.4 ± 16.3	75.5 ± 14.6
Height (cm)	174.2 ± 9.7	174.1 ± 9.3	174.0 ± 8.7
BMI (kg/m^2^)	25.8 ± 3.8	25.6 ± 5.6	24.8 ± 4.1
Physical activity			
Sedentary (n)	8 (20.0)	8 (13.3)	7 (14.6)
Moderately active (n)	23 (57.5)	30 (50.0)	31 (64.6)
Vigorously active (n)	9 (22.5)	22 (36.7)	10 (20.8)
Alcohol drinkers (n)	31 (77.5)	49 (81.7)	38 (79.2)
Smokers (n)	4 (10.0)	5 (8.3)	5 (10.4)
Salt intake (g/day) ^c^	9.1 ± 2.9	8.9 ± 2.4	9.1 ± 2.9
Sodium/potassium excretion ratio ^c,d^	2.2 [1.8,2.6]	2.3 [1.9,2.7]	2.2 [1.8,2.8]

^a^ Values are mean ± SD or n (%). Eleven participants did not complete the baseline questionnaire and therefore had missing data on smoking status, alcohol intake, physical activity and education, and six participants had missing values for BMI. BMI = body mass index. ^b^ This group also included vocational educations; shorter courses and no further education after primary-, secondary- or upper secondary school. ^c^ Five participants at baseline had missing data on salt and potassium intake. Values are based on a single 24-h urine collection for 158 participants, the average of two collections for one participant and the average of three collections for 145 participants. ^d^ Values were log transformed before analysis and mean differences and 95% CI are presented in %.

**Table 2:** Development in cardiovascular risk factors from baseline to follow-up in each group with *p*-value for change over time in each group.

	Control			Intervention Group A			Intervention Group B		
	Baseline	Follow-Up	*p*-Value	Baseline	Follow-Up	*p*-Value	Baseline	Follow-Up	*p*-Value
	N = 101			N = 81			N = 127		
Systolic BP (mmHg)	106 (12)	101 (9)	0.0103	107 (9)	103 (8)	0.0277	106 (14)	102(12)	0.0142
Diastolic BP (mmHg)	66 (6)	62 (6)	<0.0001	66 (5)	64 (6)	0.0699	67 (9)	64 (6)	0.0216
BMI (kg/m^2^)	16.42 (14.73–18.13)	16.13 (14.57–18.65)	0.9529	17.64 (15.54–19.29)	16.82 (15.04–19.26)	0.2875	16.34 (15.51–19.21)	16.29 (15.17–18.98)	0.7830
**Adults (>18 years)**									
Diastolic BP (mmHg)	77.40 (10.83)	75.40 (9.97)	0.0183	79.71 (9.50)	77.86 (7.73)	0.0488	78.38 (9.85)	75.41 (9.47)	0.0006
Systolic BP (mmHg)	120.58 (16.31)	116.69 (13.97)	0.0041	124.41 (14.29)	121.45 (10.81)	0.0407	123.16 (13.22)	116.93 (15.95)	<0.0001
Plasma total cholesterol (mmol/L)	4.49 (0.77)	4.46 (0.85)	0.7596	4.31 (0.90)	4.17 (0.77)	0.0007	4.49 (0.99)	4.32 (0.96)	0.0211
Plasma triglycerides ** (mmol/L)	0.86 (0.66–1.30)	0.92 (0.61–1.28)	0.2995	0.89 (0.63–1.26)	1.03 (0.66–1.32)	0.7308	1.04 (0.67–1.56)	0.98 (0.67–1.60)	0.3346
Plasma HDL cholesterol ** (mmol/L)	1.22 (1.04–1.40)	1.20 (1.02–1.47)	0.7278	1.15 (1.04–1.33)	1.12 (1.03–1.32)	0.2209	1.15 (0.96–1.40)	1.20 (0.99–1.32)	0.5863
Plasma LDL cholesterol ** (mmol/L)	2.70 (2.40–3.20)	2.80 (2.10–3.10)	0.1375	2.70 (2.10–3.10)	2.30 (2.00–3.00)	0.0042	2.60 (2.20–2.90)	2.30 (2.00–2.90)	0.0252
Plasma VLDL cholesterol ** (mmol/L)	0.40 (0.30–0.60)	0.40 (0.30–0.60)	0.4201	0.40 (0.30–0.60)	0.50 (0.30–0.60)	0.8809	0.50 (0.30–0.70)	0.40 (0.30–0.70)	0.3418
Plasma Aldosterone ** pmol/L	190.00 (103–261)	150.00 (103–270)	0.9338	124.00 (103–201)	138.00 (103–202)	0.8742	122.00 (103–217)	147.00 (103–222)	0.4465
Plasma Renin ** IU/L	16.50 (9.85–22)	17.00 (11–25)	0.0312	13.00 (8.00–18)	12.00 (7.75–21)	0.7128	12.00 (7.90–19)	15.00 (12–25)	0.0012
Plasma Metanephrine (adrenaline) nmol/L	0.11 (0.08)	0.12 (0.08)	0.4116	0.12 (0.05)	0.10 (0.06)	0.0225	0.12 (0.07)	0.12 (0.09)	0.3920
Plasma Normetanephrine (noradrenaline) nmol/L	0.25 (0.14)	0.21 (0.08)	0.0435	0.31 (0.11)	0.21 (0.09)	<0.0001	0.32 (0.13)	0.22 (0.11)	<0.0001
Plasma-glucose ** (mmol/L)	5.10 (4.90–5.40)	5.10 (4.90–5.40)	0.7877	5.10 (4.90–5.50)	5.20 (4.90–5.50)	0.2934	5.20 (4.80–5.40)	5.15 (5.00–5.50)	0.2586
HbA1c (mmol/mol)	34.35 (2.25)	34.81 (2.42)	0.0069	33.90 (3.00)	34.94 (3.10)	0.0021	34.23 (3.92)	34.39 (4.38)	0.4874
BMI (kg/m^2^) **	24.77 (21.76–26.99)	24.34 (21.57–27.28)	0.1599	26.01 (23.07–27.99)	25.98 (23.19–28.49)	0.6562	24.38 (22.16–27.44)	23.87 (22.06–27.04)	0.7070
Fat percent (%)	26.61 (9.59)	26.35 (8.58)	0.6193	28.39 (9.91)	27.13 (9.88)	0.0004	27.37 (10.55)	26.74 (10.79)	0.0366

Data are mean (std) or median (IQR) at baseline and follow-up in each group with *p*-value for change over time in each group. Mean/Median at baseline and follow-up are based on observed data. *p*-value from test in data with multiple imputation. ** Median (IQR). *p*-value based on test for logarithm transformed variable.

**Table 3:** Between group differences and 95% confidence interval for cardiovascular risk factors (multiple imputation).

		Intervention A Versus Control			Intervention B Versus Control			Intervention B Versus A		
	N	Difference (95% CI)		*p*-Value	Difference (95% CI)		*p*-Value	Difference (95% CI)		*p*-Value
**Children < 18 years**										
Systolic BP (mmHg)	124	0.115	(−4.469–4.700)	0.9607	−0.178	(−4.253–3.897)	0.9317	−0.293	(−4.875–4.288)	0.9001
Diastolic BP (mmHg)	124	1.282	(−1.598–4.162)	0.3829	1.643	(−0.865–4.151)	0.1991	0.361	(−2.504–3.227)	0.8047
BMI ** (kg/m^2^)	137	−0.026	(−0.067–0.015)	0.2124	0.001	(−0.035–0.038)	0.9359	0.027	(−0.015–0.069)	0.2001
**18+ years**										
Diastolic BP (mmHg)	141	−0.116	(−2.842–2.611)	0.9338	−0.807	(−3.254–1.640)	0.5179	−0.692	(−3.312–1.929)	0.6049
Systolic BP (mmHg)	141	0.729	(−3.918–5.377)	0.7584	−1.497	(−5.690–2.695)	0.4839	−2.227	(−6.657–2.204)	0.3246
Total cholesterol (mmol/L)	137	−0.251	(−0.509–0.008)	0.0574	−0.117	(−0.334–0.100)	0.2915	0.134	(−0.114–0.382)	0.2897
Triglycerides ** (mmol/L)	137	−0.030	(−0.255–0.195)	0.7950	−0.040	(−0.227–0.148)	0.6784	−0.010	(−0.233–0.213)	0.9306
HDL cholesterol ** (mmol/L)	137	−0.035	(−0.172–0.102)	0.6156	0.016	(−0.090–0.122)	0.7671	0.051	(−0.081–0.183)	0.4466
LDL cholesterol ** (mmol/L)	135	−0.099	(−0.264–0.065)	0.2360	−0.033	(−0.159–0.093)	0.6053	0.066	(−0.091–0.223)	0.4078
VLDL cholesterol ** (mmol/L)	135	−0.037	(−0.278–0.204)	0.7651	−0.047	(−0.249–0.156)	0.6521	−0.010	(−0.233–0.214)	0.9312
Aldosterone ** (pmol/L)	137	−0.054	(−0.265–0.158)	0.6196	−0.020	(−0.208–0.168)	0.8362	0.034	(−0.169–0.236)	0.7441
Renin ** (IU/L)	137	−0.193	(−0.388–0.003)	0.0535	0.040	(−0.135–0.214)	0.6568	**0.232**	**(0.045–0.420)**	**0.0152**
Metanephrine (nmol/L)	112	−0.017	(−0.059–0.025)	0.4305	−0.008	(−0.049–0.033)	0.7001	0.009	(−0.037–0.055)	0.6999
Normetanephrine (nmol/L)	112	−0.023	(−0.085–0.040)	0.4753	−0.005	(−0.061–0.050)	0.8538	0.018	(−0.045–0.080)	0.5814
Plasma-glucose ** (mmol/L)	135	0.023	(−0.023–0.068)	0.3260	0.007	(−0.032–0.046)	0.7390	−0.016	(−0.061–0.029)	0.4840
HbA1c (mmol/mol)	137	0.654	(−0.076–1.384)	0.0793	−0.318	(−0.971–0.335)	0.3400	**−0.972**	**(−1.689–0.255)**	**0.0079**
BMI ** (kg/m^2^)	142	0.004	(−0.010–0.018)	0.5572	0.008	(−0.004–0.019)	0.2155	0.003	(−0.009–0.016)	0.5975
Fat percent (%)	142	**−1.314**	**(−2.399–0.229)**	**0.0177**	−0.706	(−1.666–0.255)	0.1498	0.608	(−0.431–1.647)	0.2515

Comparisons were calculated using mixed models adjusted for baseline and with family as random effect and using multiple imputation. ** Difference (95% CI) for logarithm transformed variable. Bold indicates significant differences.

**Table 4:** Difference between-groups and 95% confidence interval for cardiovascular risk factors (complete cases).

		Intervention A Versus Control			Intervention B Versus Control			Intervention B Versus A		
	N	Difference (95% CI)		*p*-Value	Difference (95% CI)		*p*-Value	Difference (95% CI)		*p*-Value
**Children < 18 years**										
Diastolic BP (mmHg)	124	1.243	(−1.182–3.669)	0.3082	1.540	(−0.679–3.759)	0.1693	0.296	(−2.135–2.727)	0.8078
Systolic BP (mmHg)	124	−0.615	(−4.116–2.886)	0.7273	0.214	(−2.991–3.418)	0.8946	0.829	(−2.662–4.321)	0.6376
BMI ** (kg/m^2^)	137	−0.014	(−0.043–0.015)	0.3409	0.005	(−0.021–0.031)	0.7160	0.019	(−0.010–0.047)	0.1924
**18+ years**										
Diastolic BP (mmHg)	141	−0.138	(−2.640–2.363)	0.9127	−1.129	(−3.412–1.154)	0.3276	−0.991	(−3.385–1.404)	0.4122
Systolic BP (mmHg)	141	0.923	(−3.278–5.124)	0.6629	−1.770	(−5.616–2.077)	0.3622	−2.693	(−6.720–1.335)	0.1868
Total cholesterol (mmol/L)	**137**	**−0.286**	**(−0.495–0.077)**	**0.0079**	−0.151	(−0.339–0.036)	0.1122	0.135	(−0.065–0.335)	0.1835
Triglycerides ** (mmol/L)	137	−0.065	(−0.228–0.097)	0.4271	−0.065	(−0.213–0.082)	0.3800	−0.000	(−0.157–0.157)	0.9984
HDL cholesterol ** (mmol/L)	137	−0.029	(−0.088–0.030)	0.3371	0.006	(−0.047–0.059)	0.8191	0.035	(−0.022–0.092)	0.2253
LDL cholesterol ** (mmol/L)	135	**−0.076**	**(−0.148–0.003)**	**0.0413**	−0.034	(−0.098–0.031)	0.3065	0.042	(−0.027–0.111)	0.2296
VLDL cholesterol ** (mmol/L)	135	−0.097	(−0.258–0.065)	0.2366	−0.079	(−0.223–0.066)	0.2833	0.018	(−0.138–0.174)	0.8162
Aldosterone ** (pmol/L)	137	−0.084	(−0.281–0.113)	0.3982	−0.071	(−0.248–0.106)	0.4277	0.013	(−0.174–0.200)	0.8894
Renin ** (IU/L)	137	**−0.231**	**(−0.414–0.049)**	**0.0134**	0.007	(−0.157–0.171)	0.9319	**0.238**	**(0.064–0.412)**	**0.0076**
Metanephrine (nmol/L)	112	**−0.031**	**(−0.056–0.007)**	**0.0131**	−0.011	(−0.032–0.010)	0.2891	0.020	(−0.004–0.044)	0.0943
Normetanephrine (nmol/L)	112	−0.033	(−0.074–0.009)	0.1252	−0.010	(−0.046–0.026)	0.5784	0.022	(−0.018–0.062)	0.2660
Plasma-glucose ** (mmol/L)	135	0.013	(−0.021–0.047)	0.4386	0.007	(−0.023–0.038)	0.6280	−0.006	(−0.038–0.027)	0.7193
HbA1c (mmol/mol)	137	0.641	(−0.056–1.338)	0.0708	−0.382	(−1.003–0.240)	0.2254	**−1.023**	**(−1.690–0.356)**	**0.0031**
BMI ** (kg/m^2^)	142	0.005	(−0.008–0.017)	0.4698	0.008	(−0.004–0.019)	0.1908	0.003	(−0.009–0.015)	0.6280
Fat percent (%)	142	**−1.527**	**(−2.512–0.543)**	**0.0027**	−0.765	(−1.655–0.125)	0.0908	0.762	(−0.185–1.709)	0.1132

Comparisons were calculated using mixed models adjusted for baseline and with family as random effect, including only complete cases. Complete cases are defined as participants with at least one measurement at baseline and follow-up. Analyses are adjusted for sex, age, BMI, education and physical activity. Analyses for blood pressure and BMI among all participants are furthermore adjusted for alcohol intake. ** Difference (95% CI) for logarithm transformed variable. Bold indicates significant differences.

**Table 5:** Subgroup analyses of changes in cardiovascular risk factors among participants with per protocol changes in salt intake and salt/potassium ratio (participants 18+ years).

		A Reduction in Salt Intake from Baseline to Follow-Up of at Least 3 g per day (N = 31)				A Reduction in the Sodium to Potassium Ratio of at Least 20 Percent from Baseline to Follow-Up (N = 33)		
	N	Baseline	Follow-up	*p*-Value	N	Baseline	Follow-up	*p*-Value
Systolic BP (mmHg)	**30**	**123 (11)**	**115 (11)**	**<0.0001**	**32**	**123 (11)**	**117 (12)**	**<0.0001**
Diastolic BP (mmHg)	**30**	**78 (8)**	**76 (8)**	**0.0336**	**32**	**80 (8)**	**77 (9)**	**0.0212**
Plasma total cholesterol (mmol/L)	**30**	**4.48 (1.04)**	**4.28 (0.89)**	**0.0296**	**32**	**4.48 (1.04)**	**4.22 (0.97)**	**0.0005**
Plasma triglycerides ** (mmol/L)	30	1.04 (0.66–1.32)	0.89 (0.62–1.12)	0.0794	32	1.07 (0.65–1.42)	0.83 (0.62–1.14)	0.0993
Plasma HDL cholesterol ** (mmol/L)	30	1.23 (0.97–1.40)	1.20 (1.06–1.37)	0.5019	32	1.22 (1.00–1.39)	1.14 (1.04–1.32)	0.9353
Plasma LDL cholesterol ** (mmol/L)	30	2.50 (2.10–3.20)	2.50 (2.00–3.20)	0.1503	**31**	**2.40 (2.00–3.20)**	**2.30 (2.00–3.20)**	**0.0283**
Plasma VLDL cholesterol ** (mmol/L)	30	0.50 (0.30–0.60)	0.40 (0.30–0.50)	0.1071	**31**	**0.50 (0.30–0.60)**	**0.40 (0.30–0.50)**	**0.0375**
Plasma Aldosterone ** (pmol/L)	30	119.50 (103–231)	127.00 (103–242)	0.6131	32	123.00 (103–286)	158.00 (103–262)	0.4074
Plasma Renin ** (IU/L)	30	10.50 (6.80–18)	13.00 (7.80–18)	0.3039	32	10.50 (6.60–18)	13.50 (7.80–18)	0.0710
Plasma Adrenalin (nmol/L)	**23**	**0.12 (0.07)**	**0.10 (0.08)**	**0.0412**	24	0.12 (0.08)	0.11 (0.08)	0.2433
Plasma Noradrenalin (nmol/L)	**23**	**0.31 (0.13)**	**0.22 (0.09)**	**0.0006**	24	0.30 (0.13)	0.21 (0.10)	0.0001
Plasma-glucose ** (mmol/L)	28	5.25 (4.80–5.50)	5.25 (4.95–5.65)	0.3402	30	5.20 (4.90–5.50)	5.25 (4.80–5.50)	0.7358
HbA1c (mmol/mol)	30	34.77 (2.86)	35.27 (2.64)	0.0961	32	34.38 (2.81)	34.84 (2.83)	0.0917
BMI ** (kg/m^2^)	31	25.35 (21–27)	25.24 (22–27)	0.9294	33	25.77 (21–27)	25.40 (21–27)	0.3039
Fat percent (%)	31	27.71 (10.33)	27.39 (10.13)	0.2355	**33**	**27.81 (9.73)**	**27.04 (9.81)**	**0.0047**

Data are mean (std) or median (IQR) at baseline and follow-up with p-value for change over time. ** Difference (95% CI) for logarithm transformed variable. Bold indicates significant differences.
